# Identification QTLs Controlling Genes for Se Uptake in Lentil Seeds

**DOI:** 10.1371/journal.pone.0149210

**Published:** 2016-03-15

**Authors:** Duygu Ates, Tugce Sever, Secil Aldemir, Bulent Yagmur, Hulya Yilmaz Temel, Hilal Betul Kaya, Ahmad Alsaleh, Abdullah Kahraman, Hakan Ozkan, Albert Vandenberg, Bahattin Tanyolac

**Affiliations:** 1 Department of Bioengineering, Faculty of Engineering, Ege University, Bornova, Izmir, Turkey; 2 Department of Field Crops, Faculty of Agriculture, Harran University, Sanlı Urfa, Turkey; 3 Department of Field Crops, Faculty of Agriculture, Cukurova University, Adana, Turkey; 4 Crop Development Centre, University of Saskatchewan, Saskatoon, Saskatchewan, Canada; University of Guelph, CANADA

## Abstract

Lentil (*Lens culinaris* Medik.) is an excellent source of protein and carbohydrates and is also rich in essential trace elements for the human diet. Selenium (Se) is an essential micronutrient for human health and nutrition, providing protection against several diseases and regulating important biological systems. Dietary intake of 55 μg of Se per day is recommended for adults, with inadequate Se intake causing significant health problems. The objective of this study was to identify and map quantitative trait loci (QTL) of genes controlling Se accumulation in lentil seeds using a population of 96 recombinant inbred lines (RILs) developed from the cross “PI 320937” × “Eston” grown in three different environments for two years (2012 and 2013). Se concentration in seed varied between 119 and 883 μg/kg. A linkage map consisting of 1,784 markers (4 SSRs, and 1,780 SNPs) was developed. The map spanned a total length of 4,060.6 cM, consisting of 7 linkage groups (LGs) with an average distance of 2.3 cM between adjacent markers. Four QTL regions and 36 putative QTL markers, with LOD scores ranging from 3.00 to 4.97, distributed across two linkage groups (LG2 and LG5) were associated with seed Se concentration, explaining 6.3–16.9% of the phenotypic variation.

## Introduction

Lentils are grown and consumed in many developing countries and are an important dietary staple because of their high protein content and nutrient density [1]. In particular, lentil is an excellent source of macro and micronutrients and trace elements [2], including Se [3]. Se is an essential micronutrient for organisms and has beneficial effects on animal and human health [4], although toxicity can occur with acute or chronic ingestion of excess Se [5]. Se is taken up from the soil by plants in two forms: inorganic (selenate and selenite) and organic (selenomethionine and selenocysteine) [6]. Both inorganic and organic forms can be good dietary sources of selenium for humans [6]. The recommended dietary allowance (RDA) of 55–200 μg of Se per day for adults is considered essential for healthy living [7]. The recommended daily intake of Se is 55μg in the US [7] and 60–75μg in the UK [8], and a single portion of cooked rice provides only 12μg of Se [9]. Low levels of dietary Se have been linked to a number of diseases. In the case of Se deficiency, serious muscle weakness [10], cardiovascular disruption [11], delayed child development [12], reduced eye health [13], early aging [14], nervous system disorders, and mental retardation [15] may be observed.

Se is integrated with selenoproteins (SPs) known as selenocystine (SeCys) residues in polypeptides [16]. Over 25 SPs have been identified in mammals [17]. SPs have antioxidant features that protect cells from free radicals [18], strengthen the immune system [19], contribute to proper thyroid gland function, and delay the aging process [20]. SPs help balance cardiovascular health by stimulating tissue flexibility and supporting heart cells [21, 22], and also decrease the effects of toxic substances [23]. Se also plays an important role in the prevention of various cancers, including prostate [24], lung [25], colorectal [26], bladder [27], and various gastrointestinal cancers [28]. Therefore, developing micronutrient-enriched crop varieties by using genetic and genomic tools is considered a promising and cost-effective approach for controlling malnutrition worldwide [29] and increasing Se concentration in lentil seed could provide additional dietary sources of daily Se uptake.

One alternative to increasing the level of Se in seed is through conventional breeding and a strategy known as biofortification [30]. The aim of biofortification is to enrich the micronutrient concentration in the edible parts of plants using biotechnological approaches in combination with plant breeding. Development of an effective biofortification strategy is a highly effective way of decreasing the cost of reducing micronutrient deficiencies in the rural population in developing countries. Lentil is a key crop in biofortification efforts as it provides fixed atmospheric nitrogen to plants and plays a role in the management of soil fertility [31].

Determining the genetic factors controlling micronutrient concentration is useful for map-based cloning and marker assisted selection (MAS). The application of molecular markers for QTL analysis has provided an effective approach to determine these genetic factors. RILs or doubled haploid populations have been used to identify QTLs associated with micronutrient concentration [32–34]. Therefore, identification QTL alleles with micronutrient efficiency, and discovery of tightly linked molecular markers for MAS selection are very important for lentil breeding.

A biofortification approach similar to that proposed for other crops can be used to develop lentil varieties that are enriched with micronutrients [8, 35]. To date, a number of QTL studies have been conducted on micronutrient accumulation in seeds of several cops. For example, Waters et al. [36] identified several QTLs for accumulation of Ca, Cu, K, Mg, Mn, P, S, and Zn in seeds of *Arabidopsis thaliana*; Blair et al. [37] detected 26 QTLs for Fe and Zn concentration in seeds of common bean; Tiwari et al. [34] reported two QTLs for Fe and one QTL for Zn in wheat; Sankaran et al. [38] identified 46 QTLs for mineral concentration (Ca, Cu, Fe, K, Mg, Mn, P, and Zn) in *Medicago truncatula*; Tezuka et al. [39] identified three QTLs for Cd accumulation in rice; Norton et al. [40] detected six QTLs for Se concentration in rice; and Pu et al. [29] identified five QTLs associated with Se concentration using composite interval mapping in two different RILs of wheat. However, no studies have been performed to identify QTL controlling Se uptake in lentil seed. Thus, the aims of this study were to (i) determine phenotypic variation among a RIL population and (ii) identify DNA markers linked to the gene(s) controlling Se uptake in lentil seed employing a QTL mapping approach.

## Materials and Methods

### Soil analysis

Soil analyses were carried out in the Department of Plant and Soil Science at Ege University in Turkey. Soils were sampled from three experimental fields in Turkey (located at Izmir, Adana, and Sanli Urfa) to determine structural and chemical properties of the soil. Analyses were conducted for soil pH [41], total soluble salt [42], texture [43], organic matter [41], and CaCO_3_ [44]. Macro and micronutrient analyses of soil samples were carried out according to Bingham [45], Pratt [46], and Linsday and Novell [47]. Total Se concentration of experimental soils was determined as the mean of three replicates with standard error by inductively coupled plasma mass spectrometry (ICP-MS).

### Plant material and DNA extraction

The experiment was carried out on a population of 96 RILs of LR-39, which was generated from the cross between “PI 320937” (P1) × “Eston” (P2) developed at the University of Saskatchewan, Canada. The population was generated by advancing F_1_ plants from the simple cross to the F_2_ generation and then through single-seed descent from the F_2_ to the F_7_ generation. The LR-39 population was planted with three replications at each of the three field sites during the 2012 and 2013 growing seasons.

For DNA extraction, young leaves from 4–6 week old seedlings of the 96 RILs and parents were collected in aluminum foil and placed in liquid nitrogen. Leaf tissue from each individual was ground in liquid nitrogen with a tissue lyser. Then, DNA was extracted from 100 mg of fresh leaf tissue using a DNA isolation kit (Plant Genomic DNA Purification Kit Cat no: K0792 Fermentas, Germany). The purified DNA was quantified using a Qubit 2.0 Fluorometer (Invitrogen, CA, USA). The isolated DNA was kept at -86°C in a freezer until use. The stock DNA was diluted for each polymerase chain reaction (PCR) protocol (AFLP and SSR) as described below.

### Selenium analysis

Sample preparation and analyses were performed according to previously described procedures [48]. A total of 1 g of each ground sample was acid digested with HNO_3_-HClO_4_ (4:1) mixed using a heating protocol, increasing from 100 to 220°C for 2 h on a digestion block. The final solutions were diluted with ultrapure water to a final volume of 50 mL. Se concentration was determined by ICP-MS with an Agilent 7700 instrument [49]. A commercially available Se standard solution (Cat no: 1197960100, traceable to SRM from NIST SeO_2_ in HNO_3_, ρ = 1000 mg/L Se, Merck, Darmstadt, Certipur®, Germany) was used to prepare calibration curve graphs. Details of this procedure are described by Gupta [50]. Se analysis for each RIL was replicated three times.

### Variance analysis

Analysis of variance (ANOVA) was carried out using TOTEMSTAT software [51] for Se concentrations of the RILs grown in different locations and years. Probability was accepted at the P ≤ 0.001 and P ≤ 0.05 levels.

### DNA analysis with genetic markers

#### SSR analysis

For SSR analysis, each 20 μL PCR reaction contained 40 ng of genomic DNA, 10 pmol of each forward and reverse primer, 2.5 mM dNTP, 1X PCR buffer (75 mM Tris-HCl, PH: 8.8, 20 mM (NH_4_)_2_SO_4_, 25 mM MgCl_2_, 0,1% Tween), and 0.6 U Taq DNA polymerase. PCR was carried out in a thermocycler (MJ Research^TM^, Nevada, USA) with an initial denaturation of 94°C for 5 min, followed by 30 cycles of 94°C for 30 s, 55°C for 35 s, and 72°C for 60 s, followed by a final extension at 72°C for 5 min. The PCR products were resolved using 6% polyacrylamide gel electrophoresis on a LI-COR 4300 DNA analyzer (Li-COR Lincoln NE, USA). Forward primers were modified by adding an M13 tail (5ˈ-TGTAAAACGACGGCCAGT–3ˈ) and labelled with IRDye 700 or IRDye 800. The band sizes were analyzed using SagaGT software (Li-COR Lincoln, NE, USA).

#### SNP analysis

SNP analysis was performed using a genotyping by sequencing (GBS) approach. GBS analysis followed the procedure described by Raman et al. [52]. Briefly, two different restriction enzymes were used to digest 100 ng of reference DNA. *Pst* I and *Mse* I enzymes were applied to the DNA to create overhangs for adapter ligation. A staggered, varying length barcode region, the Illumina flowcell attachment, and a sequencing primer sequence were included in the *Pst* I-compatible adapter. The *Mse* I-compatible overhang sequence was included in the reverse adapter along with the flowcell attachment sequence. The following PCR protocol was performed for 30 rounds to effectively amplify only the *Pst* I/*Mse* I fragments: 94°C for 1 min, followed by 29 cycles of 94°C for 20 s, ramp at 2.4°/s to 58°C, hold at 58°C for 30 sec, ramp at 2.4°C/sec to 72°C, hold at 72°C for 45 s, extend for 7 min at 72°C, and then finally cool to 10°C. To sequence on the Illumina Hiseq2000, the PCR products were multiplexed in equimolar amounts and a c-Bot (Illumina) bridge PCR was applied. A single read was run for 77 cycles. In a single lane, all amplicons were sequenced and analyzed by proprietary DArT analytical pipelines. To filter away poor quality sequences, the FASTQ files were first processed in the primary pipeline. A Phred pass score of 30 was chosen for the barcode region as a more stringent selection than for the rest of the sequence. Thus, in the barcode split step, very reliable results were obtained during assignment of the sequences to specific samples. In marker calling, approximately 2,000,000 sequences per barcode/sample were identified and used. In the secondary pipeline, these files were used for DArT P/L’s proprietary SNP and Presence/Absence Markers (PAM) calling algorithms (DArTsoftseq). The analytical pipeline processed the sequence data. The parental lines (“PI 320937” × “Eston”) of the LR-39 RIL population generated all polymorphic sequences for the DArT-Seq markers. SNP data was uploaded system as S1 Supporting Information.

### Genetic linkage mapping and QTL analysis

Polymorphic bands were scored for each RIL individual and recorded as either type ‘A’ (“PI 320937”) or ‘B’ (“Eston”), evaluated for the presence or absence of bands, and later combined to construct a genetic linkage map. All genotypic marker data were tested for segregation distortion using JoinMap4.0 linkage map software [53]. Distorted markers were eliminated and not used for linkage mapping. Linkage analysis was performed using maximum likelihood mapping algorithm with RIL population type, using Kosambi function, logarithm of the odds (LOD) of 2.0–10.0, and a recombination fraction of 0.35 as mapping parameters. The genotypic and phenotypic data sets were imported into MapQTL software for QTL analyses [54]. The QTL were determined following Simple Interval Mapping (SIM) [55]. The significant LOD scores for detection of the QTL were calculated based on 1000 permutations at P ≤ 0.05 and 0.01 [53]. The proportion of observed genetic variation explained due to a particular QTL was estimated by the coefficient of determination (*R*^*2*^) using maximum likelihood for SIM.

## Results

### Total Se concentration and phenotypic variation in seeds of lentil genotypes

**Fig 1:**
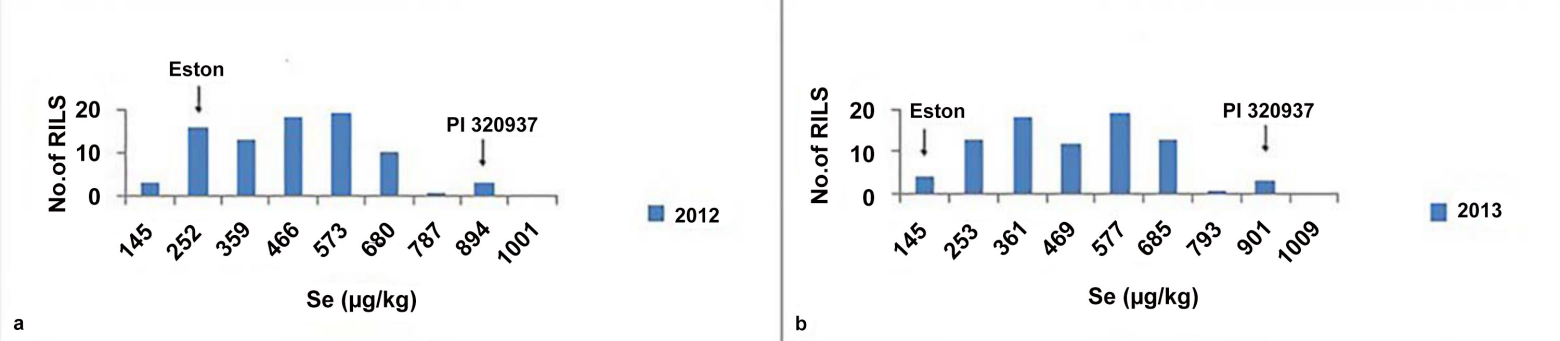
Histograms of seed Se concentration in the lentil RIL population for (a) 2012 and (b) 2013. (a) distribution of lentil seed Se concentration in 2012 and (b) distribution of lentil seed Se concentration in 2013.

Mean Se concentration for the RIL parents, “Eston” and “PI 320937”, were 166 and 858 μg/kg, respectively, across the two study years and three growing locations (Table 1). Mean seed Se concentration within the RIL population ranged from 92 to 908 μg/kg (mean 421 μg/kg), and was very consistent across the three growing locations and over both study years (Table 1). Se concentrations in lentil seed from the LR-39 RIL population were normally distributed (Fig 1A and 1B). Soil analyses for Adana, Izmir, and Sanli Urfa indicate high levels of Se at Adana (89 μg/kg) and Sanli Urfa (53 μg/kg) and adequate levels at Izmir (29 μg/kg) (Table 1).

**Table 1:** Total Se concentrations for both years and three locations in the RIL population.

Se concentration (μg/kg)							
	Izmir		Adana		S. Urfa		
Parents	2012	2013	2012	2013	2012	2013	Mean
Eston	189	115	178	148	202	169	166
PI 320937	889	692	908	890	876	893	858
**RIL population**							
Minimum	104	144	117	115	145	92	119
Maximum	889	890	908	870	876	867	883
Mean	420	408	416	426	424	431	421
**Soil**	29		89		53		

The ANOVA for Se concentration for all environments and years (Table 2) shows that genotype, location, year, and the interactions between year × genotype and location × genotype were highly significant (P ≤ 0.001). The interaction between year × location was not significant. Significant variation was detected among the RILs for Se concentration at the three locations and in the two years at P ≤ 0.001.

**Table 2:** Summary of ANOVA for total Se concentration of RILs.

Variation source	Degree of freedom	Mean square	F	F probability
Year	1	34341.4	81.1 **	10.89
Location	2	4077.5	9.6 **	6.92
Genotype	80	545521.0	1289.5 **	1.62
Year × Location	2	607.4	1.4 ns	6.92
Year × Genotype	80	1487.0	3.5 **	1.62
Location × Genotype	160	820.3	1.9 **	1.48
Year × Location × Genotype	160	876.8	2.0 **	1.48
Error	970	423.0	-	-
General	1457	-	-	-

Coefficient of Variation = 4.86%

ns = Not significant.

** = Significant at P ≤ 0.001

### SSR and SNP marker analysis

**Fig 2:**
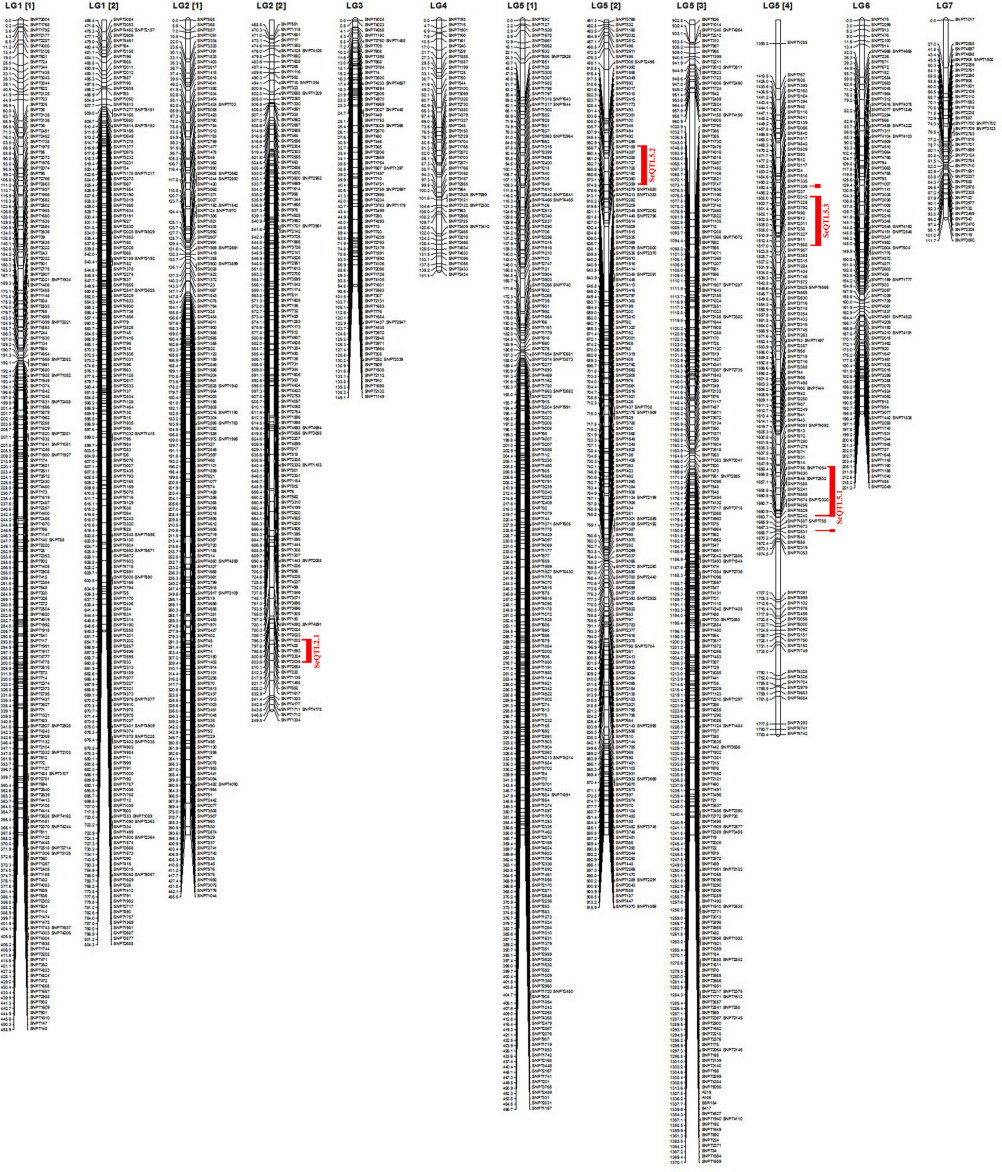
Linkage map of lentil based on SNP, and SSR. Red bars and vertical letters indicate QTL regions. Left bar of linkage map is cM distance and right bar of linkage map is marker names. Numbers in brackets indicate continue of certain linkage map such as 1[1].

To determine whether the DNA markers used in this study show segregation distortion based on the expected Mendelian ratio, segregation distortion analysis was carried out using JoinMap 4.0 software (Table 3). A total of 3,030 markers (30 SSRs, and 3000 SNPs) were analyzed for the mapping of the genome. Of these, 1,784 markers were mapped (4 SSRs, and 1,780 SNPs markers) in the genome (Fig 2), whereas 1,076 (16 SSRs, and 1,060 SNPs) markers remained unlinked. A total of 170 markers (5.6% of the polymorphic markers) showed segregation distortion.

**Table 3:** Results of JoinMap 4.0 analysis used to determine DNA marker quality.

Type of Marker	Total of polymorphic markers	Number of distorted markers	Distortion Ratio (%)	Unlinked markers	Linked markers
SSR	30	10	33.3	16	4
SNP	3,000	160	5.3	1,060	1,780
SSR+SNP	3,030	170	5.6	1,076	1,784

### Linkage mapping results

The map spanned a total length of 4,060.6 cM and composed of seven LGs with an average of 2.3 cM between adjacent markers (Fig 2). The longest LG was LG5 (1,783.4 cM) and the shortest was LG7 (111.7 cM). Average marker distance varied by LG; the smallest distance between markers was noted for LG3 (1.8 cM) and the greatest for LG4 (2.7 cM). SNPs were distributed throughout all LGs; however, SSRs were only mapped on LG5, which contained the most markers (782) with an average distance between markers of 2.3 cM. LG7 contained the fewest markers (42) with an average distance between markers of 2.6 cM (Table 4).

**Table 4:** Summary of the marker distribution on different linkage groups of the lentil genome.

Linkage groups	Length (cM)	Number of markers (%)	Average distance between markers (cM)	Number of SSRs	Number of SNPs	LOD
LG 1	804.3	403 (22.6)	1.9	-	403	7
LG 2	849.5	323 (18.1)	2.6	-	323	7
LG 3	149.1	81 (4.5)	1.8	-	81	7
LG 4	141.4	52 (2.9)	2.7	-	52	7
LG 5	1,783.4	782 (43.8)	2.3	4	778	6
LG 6	221.2	101 (5.7)	2.2	-	101	5
LG 7	111.7	42 (2.3)	2.6	-		4
**Total**	**4,060.6**	**1784**	**2.3**	**4**	**1780**	

### QTL analysis results

A total of four QTL regions identified using SIM were detected for Se concentration in lentil seeds for the LR-39 population and deemed to be of significant importance (Fig 2). LG2 contained only one QTL region whereas LG5 contained three QTLs for Se concentration. The QTL regions for Se located on LG2 and LG5 showed clusters of SNP markers. However, SSR markers did not locate on these regions. A total of 36 putative QTL markers were detected. The markers were only statistically significant in one year and/or in two locations. Because they did not have a stable LOD threshold (some were below LOD 3 in some years and in some locations), they can be considered putative QTL. Four QTL regions and 36 putative QTL markers, with LOD scores ranging from 3.00 to 4.97, were distributed across LG2 and LG5 and were associated with seed Se concentration, explaining 6.3–16.9% of the phenotypic variation (Table 5).

**Table 5:** QTL regions for Se concentrations in seeds of the RILs.

**QTL**	**Peak at cM**	LG	Position cM	Coverage cM	Number of markers in the QTL region	% Explanation	LOD (2012)			LOD (2013)		
							Adana	Izmir	Sanli Urfa	Adana	Izmir	Sanli Urfa
**SeQTL2.1**	796.3	2	796.3–803.6	7.3	5	6.6–11.5	3.24	3.28	3.26	3.38	3.96	3.26
**SeQTL5.2**	565.6	5	556.7–574.8	18.1	8	6.3–10.3	3.24	3.65	3.54	3.75	3.54	3.68
**SeQTL5.3**	1510.9	5	1500.0–1517.0	17.0	10	6.4–10.0	3.68	3.51	3.57	3.54	3.16	3.40
**SeQTL5.1**	1658.5	5	1656.4–1663.7	7.3	13	6.5–16.9	3.90	3.71	3.60	3.67	3.80	3.67

In the current study, LG2 contained one QTL region for Se concentration located between 796.3 and 803.6 cM, covering 7.3 cM and explaining 6.6–11.5% of the phenotypic variance. The QTL region (SeQTL2-1) on LG2 (Fig 2) clustered with five SNPs. LG5 contained three QTL regions for Se concentration. Two QTL regions (SeQTL5-1 and SeQTL5-3; Fig 2) for Se concentration were found close to each other, located between 1500.0 and 1517.0 cM and 1656.4 and 1663.7 cM and explaining 6.4–10.0% and 6.5–16.9% of the phenotypic variance, respectively. SeQTL5-1 clustered with 13 SNPs covering 7.3 cM. SeQTL5-2 (Table 5 and Fig 2) was located between 556.7 and 574.8 cM and explained 6.3–10.3% of the phenotypic variance. SeQTL5-2 (Table 5 and Fig 2) clustered with 8 SNPs covering 18.1 cM.

## Discussion

Breeding for improved Se uptake by lentil may be an effective and sustainable strategy to address micronutrient malnutrition in the long term [56]. QTL studies have been carried out on a number of staple crops to determine micronutrient accumulation in seeds (e.g., [36, 40]). The present work aimed to add to the literature in this regard for Se in lentil, and further bridge the gap between agriculture and human health.

In the current study, mean Se concentrations of LR-39 parents (“Eston” × “PI 320937”) were 166 and 858 μg/kg, respectively, while the range for the RIL population was 119 to 883 μg/kg. Rahman et al. [56] reported similar results for lentil seeds grown in Bangladesh, with total Se concentrations ranging from 74 to 965 μg/kg (mean 312 μg/kg). Thavarajah et al. [3] report Se concentrations of 425–673 μg/kg for lentil grown in Canada, which also falls within the range of results obtained here. Se concentrations for lentil seed determined in the current study are higher than values determined for other crops, including wheat [4], rice [40], broccoli [57] and soybean [58].

The significant effects noted here for genotype, years, location, and interactions between year × genotype and location × genotype agree with results reported by Thavarajah et al. [16]. Rahman et al. [56] also report that location × genotype effects were significant for seed Se in lentil; however, year × location was not significant in the present study. The lack of significance in the year × location interaction for seed Se can be attributed to the differences between genotypes being consistent across the three locations for two years.

In the current study, a total of 170 markers (5.6% of the polymorphic markers) showed segregation distortion. Similar levels of segregation distortion have been detected in several lentil mapping studies [31, 59, 60, 61]. Segregation distortion can be explained by many factors, such as small chromosomal rearrangements with little impact on fertility, chromosome pairing during meiosis [62], abortion of the male or female gametes or zygotes, or linkage to a lethal allele in gametes [63, 64]. Chromosomal rearrangements may also lead to segregation distortion [65].

The current map covers 4,060.6 cM and is composed of seven linkage groups, which corresponds to the haploid chromosome number (2n = 2x = 14) of lentil [59, 66, 67]. Previous studies with lentil using a low number of markers obtained several LGs [31, 59, 60, 61, 67]; however, genome coverage in this study is far higher than many earlier efforts (e.g., 751 cM [59]; 784.1 cM [60]; 834.7 cM [66]; 1396.3 cM [31]; 2172.4 cM [61]; 3843.4 cM [67]). SNP markers were not distributed evenly among LGs and we detected different in length of LGs. Similarly unequal marker distribution among LGs (between 58 and 226cM) was also found by Sharpe et al (2013) [66]. This could be due to variation among physical length of lentil chromosomes. Supporting our results, several karyotype analysis of lentil showed that the physical length of lentil chromosomes showed variation between 8.59μm (chromosome (chr) 1) and 5.59μm (chr7) (Salimuddin and Ramesh 1994; Gaffarzade-Namazi et al 2007; Balyan et al 2002). LG 5 was the longest LG in the current study could be chr 1 according to Gaffarzade-Namazi et al (2007); Balyan et al (2002)’ results.

Among the previously published reports, Sharpe et al. [66]’s number of markers was the highest to date for lentil linkage mapping and was identified a large collection of SNPs and subsequently developed a genotyping platform to establish the first comprehensive genetic map of the *L*. *culinaris* genome. In an LR-18 population of lentil, they mapped six SSRs and 454 SNPs, covering the genome in 834.7 cM. The lentil genome is ~4 GB in size [31], and therefore our genome coverage is closer to the size of the lentil genome than maps developed in other studies. The average distance between markers in the current map is 2.3 cM, which is shorter than reported for previous maps (6.87 cM [60]; 8.4 cM [31]; 15.87 cM [61]; and 19.3 cM [67]) and close to that reported by Sharpe et al. (1.67 cM) [66].

A number of QTL studies have been conducted to date on micronutrient accumulation in seeds of several crops [29, 32, 33, 34, 36, 40, 58]. In the current study, four QTL regions and 36 putative QTL markers controlling Se uptake in lentil seed were identified. LOD scores ranging from 3.00 to 4.97 and distributed across two LGs (LG2 and LG5) were associated with seed Se concentration, explaining 6.3–16.9% of the phenotypic variation. These phenotypic variation results are higher than for Norton et al.’s study of rice [40] and Ramamurty et al.’s study of soybean [58]. QTL results are similar to studies in other crops, in which Norton et al. [40] identified six QTLs for Se concentrations in rice and Pu et al. [29] identified four QTL regions associated with Se concentration in wheat. In Pu et al.’s study [29], one QTL region for Se concentration located between 129.2 and 161.7 cM explained 23.4% of the phenotypic variance and clustered with three markers covering 32.5 cM. Three QTL regions located between 25.8 and 35.9 cM, 121.7 and 141.2 cM, and 159.2 and 165.9 cM on different chromosomes explained 6.4, 10.1, and 28.5% of the phenotypic variance, respectively. Overall, these studies show that a high level of genetic variation and the use of more individuals can help resolve QTL regions and also allow for the characterization of regions with high LOD scores.

### Conclusions

Large phenotypic variation was obtained among lentil RILs in terms of Se accumulation in seed. However, the use of molecular markers for developing maps and other breeding applications has been limited in lentil by the low genetic variation at the species level and the lack of available marker resources. This study addressed the shortcomings of using a large number of SNP markers and reports the first results using QTL analysis for Se accumulation in lentil seed. Data from the current study may help to advance molecular breeding techniques that would allow breeders to develop varieties with higher seed Se concentrations. The use of GBS technology in lentil showed that the marker system could be promising for mapping the lentil genome at a higher density. The markers can be used for QTL analysis. Construction of a linkage map of lentil using a RIL population with variation for Se concentration in seeds enabled detection of potential QTLs controlling Se accumulation. This study also showed that Se concentration in seed was quantitatively inherited. Because RIL LR-39-98 contained the highest Se concentration, it could be used as a donor parent in breeding programs to develop Se-biofortified varieties.

## Supporting Information

S1 Supporting Information SNP linkage mapping data. (XLSX)

## References

[1] Anoma A, Collins R, McNeil D. The value of enhancing nutrient bioavailability of lentils: the Sri Lankan Scenario. African Journal Food, Agriculture, Nutrient and Development 2014; 14(7): 9529–9543.

[2] Wang N, Daun JK. Effects of variety and crude protein content on nutrients and anti-nutrients in lentils (*Lens culinaris*). Food Chem 2006; 95(3): 493–502.

[3] Thavarajah D, Thavarajah P, Wejesuriya A, Rutzke M, Glahn RP, Combs GF Jr et al The potential of lentil (*Lens culinaris* L.) as a whole food for increased selenium, iron, and zinc intake: Preliminary results from a 3 years study. Euphytica 2011; 180: 123–128.

[4] Adams ML, Lombi E, Zhao FJ, McGrath SP. Evidence of low selenium concentrations in UK bread-making wheat grain. J Sci Food Agric 2002; 82: 1160–1165.

[5] Ellis DR, Salt DE. Plants, selenium and human health. Curr Opin Plant Biol 2003; 6: 273–279. 12753978 10.1016/s1369-5266(03)00030-x

[6] Cakır O, Turgut-Kara N, Ari S. Selenium metabolism in plants: Molecular approaches. Advances in selected plant physiology aspects. ISBN 978-953-51-0557-2. Published in print edition April, 2012.

[7] Monsen ER. Dietary reference intakes for the antioxidant nutrients: vitamin C, vitamin E, selenium, and carotenoids. J Amer Diet Assoc 2000; 100: 637–640. 10863565 10.1016/S0002-8223(00)00189-9

[8] Broadley MR, White PJ, Bryson RJ, Meacham MC, Bowen HC, Johnson SE et al Biofortification of UK food crops with selenium. Proc. Nutr. Soc. 2006; 65: 169–181. 16672078 10.1079/pns2006490

[9] U.S. Department of Agriculture, Agricultural Research Service. USDA National Nutrient Database for Standard Reference, Release 21. Nutrient Data Laboratory Home Page, 2008. Available: http://www.ars.usda.gov/ba/bhnrc/ndl. Accessed 28 Jan 2009.

[10] Gore F, Fawell J, Bartram J. Too much or too little? A review of the conundrum of selenium. World Health Organization 2010. J Water Health 08.03.2010. 10.2166/wh.2009.060 20375470

[11] Coppinger RJ, Diamond AM. Selenium deficiency and human disease, In Hatfield DL (ed.), Selenium: its molecular biology and role in human health. Kluwer Academic Publishers, Norwell, Mass; 2001 pp. 219–233.

[12] Lawrence KC, Howard GE. Manifestations of chronic selenium deficiency in a child receiving total parenteral nutrition. Am J Clin Nutr 1983; 37: 319–328. 6401914 10.1093/ajcn/37.2.319

[13] Flett JC, Mayer J. Dietary Selenium repletion may reduce cancer incidence in people at high risk who live in areas with low soil selenium. Nutr Rev 1997; 55: 277–279. 9279064 10.1111/j.1753-4887.1997.tb01617.x

[14] Zheng JK, Zhang XW, Yang L, Zhang T. Inheritance of selenium contents and QTL detection in rice grains. Plant Genetic Resources; 2010 pp. 445–450.

[15] Pitts MW, Byrns CN, Ogawa-Wong AN, Kremer P, Berry MJ. Selenoproteins in nervous system development and function. Biol Trace Elem Res 2014; 161: 231–245. 10.1007/s12011-014-0060-2 24974905 PMC4222985

[16] Thavarajah D, Ruszkowski J, Vandenberg A. High potential for selenium biofortification of lentils (*Lens culinaris* L.). J Agric Food Chem 2008; 56: 10747–10753. 10.1021/jf802307h 18954072

[17] Kryukov GV, Castellano S, Novoselov SV, Lobanov AV, Zehtab O, Guigo R, Gladyshev VN. Characterization of mammalian selenoproteins. Science 2003; 300: 1439–1443. 12775843 10.1126/science.1083516

[18] Bordoni A, Danesi F, Malaguti M, Di Nunzio M, Pasqui F, Maranesi M et al Dietary selenium for the counteraction of oxidative damage: fortified foods or supplements? Br J Nutr 2008; 99: 191–197. 17651521 10.1017/S0007114507793911

[19] Broome CS, McArdle F, Kyle JAM, Andrews F, Lowe NM, Hart CA et al An increase in selenium intake improves immune function and poliovirus handling in adults with marginal selenium status. Am J Clin Nutr 2004; 80: 154–62. 15213043 10.1093/ajcn/80.1.154

[20] Zhang S, Rocourt C, Cheng WH. Selenoproteins and the aging brain. Mech Ageing Dev 2010; 131: 253–260. 10.1016/j.mad.2010.02.006 20219520

[21] Flores-Mateo G, Navas-Acien A, Pastor-Barriuso R, Guallar E. Selenium and coronary heart disease: a meta-analysis. Am J Clin Nutr 2006; 84: 762–73. 17023702 10.1093/ajcn/84.4.762 PMC1829306

[22] Bleys J, Navas-Acien A, Guallar E. Serum selenium levels and all-cause, cancer, and cardiovascular mortality among US adults. Arch Intern Med 2008; 168: 404–10. 10.1001/archinternmed.2007.74 18299496

[23] Navarro-Alarcon UM, Lopez-Martinez MC. Essentiality of selenium in the human body: relationship with different diseases. Sci Total Environ 2000; 249: 347–371. 10813463 10.1016/s0048-9697(99)00526-4

[24] Karunasinghe N, Ryan J, Tuckey J, Masters J, Jamieson M, Clarke LC et al DNA stability and serum selenium levels in a high-risk group for prostate cancer. Cancer Epidemiol Biomarkers Prev 2004; 13: 391–397. 15006914

[25] Della Rovere F, Granata A, Familiari D, Zirilli A, Cimino F, Tomaino A. Histamine and selenium in lung cancer. Anticancer Res 2006; 26: 2937–2942. 16886617

[26] Peters U, Takata Y. Selenium and the prevention of prostate and colorectal cancer. Mol Nutr Food Res 2008; 52(11): 1261–1272. 10.1002/mnfr.200800103 18763256 PMC3179524

[27] Kellen E, Zeegers M, Buntinx F. Selenium is inversely associated with bladder cancer risk: a report from the Belgian case-control study on bladder cancer. Int J Urol. 2006; 13(9): 1180–4. 16984549 10.1111/j.1442-2042.2006.01526.x

[28] Bjelakovic G, Nikolova D, Simonetti RG, Gluud C. Systematic review: Primary and secondary prevention of gastrointestinal cancers with antioxidant supplements. Aliment Pharmacol Ther 2008; 28: 689–70. 19145725 10.1111/j.1365-2036.2008.03785.x

[29] Pu ZE, Yu M, He QY, Chen GY, Wang JR, Liu YX et al Quantitative trait loci associated with micronutrient concentrations in two recombinant inbred wheat lines. Journal of Integrative Agriculture; 2013 pp. 2095–3119.

[30] Rayman MP. The use of high-selenium yeast to raise selenium status: How does it measure up? Br J Nutr 2004; 92: 557–573. 15522125 10.1079/bjn20041251

[31] Tanyolac B, Ozatay S, Kahraman A, Muehlbauer F. Linkage mapping of lentil (*Lens culinaris* L.) genome using recombinant inbred lines revealed by AFLP, ISSR, RAPD and some morphologic markers. J Agric Biotech Sustain Dev 2010; 2(1): 1–6.

[32] Shi RL, Li HW, Tong YP, Jing RL, Zhang FS, Zou CQ. Identification of quantitative trait locus of zinc and phosphorus density in wheat (*Triticum aestivum* L.) rain. Plant Soil 2008; 306: 95–104.

[33] Peleg Z, Cakmak I, Ozturk L, Yazici A, Jun Y, Budak H et al Quantitative trait loci conferring grain mineral nutrient concentrations in durum wheat × wild emmer wheat RIL population. Theoretical and Applied Genetics 2009; 119: 353–369. 10.1007/s00122-009-1044-z 19407982

[34] Tiwari VK, Rawat N, Chhuneja P, Neelam K, Aggarwal R, Randhawa GS et al Mapping of quantitative trait loci for grain iron and zinc concentration in diploid A genome wheat. Journal of Heredity 2009; 100: 771–776. 10.1093/jhered/esp030 19520762

[35] White PJ, Broadley MR. Biofortification of crops with seven mineral elements often lacking in human diets–iron, zinc, copper, calcium, magnesium, selenium and iodine. New Phytol 2009; 182: 49–84. 10.1111/j.1469-8137.2008.02738.x 19192191

[36] Waters BM, Grusak MA. Quantitative trait locus mapping for seed mineral concentrations in two *Arabidopsis thaliana* recombinant inbred populations. New Phytol 2008; 179: 1033–1047. 10.1111/j.1469-8137.2008.02544.x 18631293

[37] Blair MW, Astudillo C, Grusak MA, Graham R, Beebe SE. Inheritance of seed iron and zinc concentrations in common bean (*Phaseolus vulgaris* L.). Mol Breeding 2009; 23: 197–207.

[38] Sankaran RP, Huguet T, Grusak MA. Identification of QTL affecting seed mineral concentrations and content in the model legume *Medicago truncatula*. Theor Appl Genet 2009; 119: 241–253. 10.1007/s00122-009-1033-2 19396421

[39] Tezuka K, Miyadate H, Katou K, Kodama I, Matsumoto S, Kawamoto T et al A single recessive gene controls cadmium translocation in the cadmium hyperaccumulating rice cultivar Cho-Ko-Koku. Theor Appl Genet 2010; 120: 1175–1182. 10.1007/s00122-009-1244-6 20039013

[40] Norton GJ, Deacon CM, Xiong L, Huang S, Meharg AA, Price AH. Genetic mapping of the rice ionome in leaves and grain: Identification of QTLs for 17 elements including arsenic, cadmium, iron and selenium. Plant Soil 2010; 329: 139–153.

[41] Black CA. Methods of soil analysis Editor: Madison. Wisconsin, USA: American Society of Agronomy Inc; 1965 pp. 1372–1376.

[42] Richards LA. Diagnosis and improvement of saline and alkali soils. United States Department of Agriculture Handbook. USA 1954.

[43] Bouyoucos GJ. A recalibration of the hydrometer method for making mechanical analysis of the soils. Agronomy Journal 1955; 4(9): 419–434.

[44] Schlicting E, Blume HP. Bodenkundliches practicum. Editor: Parey, VP Hamburg; 1966. p. 94.

[45] Bingham FT. Soil test for phosphate. California Agriculture 1949; 3(7): 11–14.

[46] Pratt PF. Methods of soil analysis Editor: Black CA, Wisconsin, USA: American Society of Agronomy Inc; 1965 pp. 1010–1022.

[47] Linsday WL, Norvell WA. Development of a DTPA soil test for zinc, iron, manganese and copper. Soil Sci Soc of Amer Journal 1978; 42: 421–428.

[48] Kacar B. Chemical analysis of plant and soil: II. Plant analysis. Ankara University Press, Publication no: 453, Application book no: 155 Ankara, Turkey 1972.

[49] Kacar B, Inal A. Plant analyses. Nobel Press, First edition, No: 1241, Natural applied sciences, No: 63, ISBN 978-605-395-036-3 Ankara, Turkey 2008.

[50] Gupta UC. Handbook of Reference Methods for Plant Analysis. Editor: Kalra YP, Boca Raton; CRC Press 1998; 171–182.

[51] Acikgoz N, Ilker E, Gokcol A. Computer evaluations of biological research. Ege University Press, 2004, TOTEM, No: 2, Izmir, Turkey.

[52] Raman H, Raman R, Kilian A, Detering F, Carling J, Coombes N, et al Genome-wide delineation of natural variation for pod shatter resistance in *Brassica napus*. PLoS ONE 2014, 9(7): e101673 10.1371/journal.pone.0101673 25006804 PMC4090071

[53] Van Ooijen JW, Voorrips RE. JoinMap 4.0, software for the calculation of genetic linkage maps in experimental populations Plant Research International, Wageningen, the Netherlands 2004.

[54] Van Ooijen JW. MapQTL 6. Software for the mapping of quantitative trait loci in experimental populations of diploid species Kyazma BV: Wageningen, Netherlands 2009.

[55] Churchill GA, Doerge RW. Empirical threshold values for quantitative trait mapping. Genetics 1994; 138: 963–971. 7851788 10.1093/genetics/138.3.963 PMC1206241

[56] Rahman WMM, Zaman EMS, Thavarajah P, Thavarajah D, Siddique KHM. Selenium biofortification in lentil (*Lens culinaris Medikus* subsp. *culinaris*): Farmers' field survey and genotype × environment effect. Food Res Int 2013; 54: 1596–1604.

[57] Farnham MW, Hale AJ, Grusak MA, Finley JW. Genotypic and environmental effects on selenium concentration of broccoli heads grown without supplemental selenium fertilizer. Plant Breed 2007; 126: 195–200.

[58] Ramamurthy RK, Jedlicka J, Graef GL, Waters BM. Identification of new QTLs for seed mineral, cysteine, and methionine concentrations in soybean [*Glycine max* (L.) Merr.]. Mol Breeding 2014; 34: 431–445.

[59] Hamwieh A, Udupa SM, Choumane W, Sarker A, Dreyer F, Jung C et al A genetic linkage map of *Lens* spp. based on microsatellite and AFLP markers and the localization of *Fusarium vascular* wilt resistance. Theor Appl Genet 2005; 110: 669–677. 15650814 10.1007/s00122-004-1892-5

[60] Rubeena FR, Taylor PWJ. Construction of an intraspecific linkage map of lentil (*Lens culinaris* ssp. *culinaris*). Theor Appl Genetics 2003; 107: 910–916. 12830386 10.1007/s00122-003-1326-9

[61] Duran Y, Fratini R, Garcia P, Perez de la Vega M. An intersubspecific genetic map of lens. Theor Appl Genet 2004; 108: 1265–1273. 14676948 10.1007/s00122-003-1542-3

[62] Slocum MK, Figdore SS, Kennard WC, Suzuki JY, Osborn TC. Linkage arrangement of restriction fragment length polymorphism loci in *Brassica oleracea*. Theor Appl Genet 1990; 80: 57–64. 10.1007/BF00224016 24220811

[63] Pillen K, Steinruecken G, Wricke G, Herrman RG, Jung CA. Linkage map of sugar beet (*Beta vulgaris* L.). Theor Appl Genet 1992; 84: 129–135. 10.1007/BF00223992 24203039

[64] Wagner H, Weber WE, Wricke G. Estimating linkage relationship of isozyme markers and morphological markers in sugar beet (*Beta vulgaris* L.) including families with distorted segregations. Plant Breeding 1992; 108: 89–96.

[65] Paterson AH, Bowers JE, Burow MD, Draye X, Elsik CG, Jiang CX et al Comparative genomics of plant chromosomes. The Plant Cell 2000; 12:1523–1539. 11006329 10.1105/tpc.12.9.1523 PMC149067

[66] Sharpe AG, Ramsay L, Sanderson LA, Fedoruk MJ, Clarke WE, Li R et al Ancient orphan crop joins modern era: Gene-based SNP discovery and mapping in lentil. BMC Genomics 2013; 14: 192 10.1186/1471-2164-14-192 23506258 PMC3635939

[67] Gupta M, Verma B, Kumar N, Chahota RK, Rathour R, Sharma SK et al Construction of intersubspecific molecular genetic map of lentil based on ISSR, RAPD and SSR markers. J Genet 2012; 91: 279–287. 23271013 10.1007/s12041-012-0180-4

