# Correction: Identification QTLs Controlling Genes for Se Uptake in Lentil Seeds

**DOI:** 10.1371/journal.pone.0154054

**Published:** 2016-04-14

**Authors:** Duygu Ates, Tugce Sever, Secil Aldemir, Bulent Yagmur, Hulya Yilmaz Temel, Hilal Betul Kaya, Ahmad Alsaleh, Abdullah Kahraman, Hakan Ozkan, Albert Vandenberg, Bahattin Tanyolac

In the Funding section, the grant number from the funder Scientific and Technological Research Foundation of Turkey is listed incorrectly. The correct grant number is: TUBITAK-COST-111O446.
